# Amygdalar and hippocampal volume loss in limbic-predominant age-related TDP-43 encephalopathy

**DOI:** 10.1093/brain/awaf201

**Published:** 2025-06-13

**Authors:** Alex Wesseling, Ismael L Calandri, Maud M A Bouwman, Niels Reijner, Natasja A C Deshayes, Frederik Barkhof, Rik Ossenkoppele, Wilma D J van de Berg, Annemieke J M Rozemuller, Yolande A L Pijnenburg, Jeroen J M Hoozemans, Laura E Jonkman

**Affiliations:** Department of Anatomy and Neurosciences, Amsterdam UMC, Amsterdam 1081 HV, The Netherlands; Amsterdam Neuroscience, Neurodegeneration, Amsterdam 1081 HV, The Netherlands; Alzheimer Center Amsterdam UMC, Department of Neurology, Amsterdam 1081 HV, The Netherlands; Amsterdam Neuroscience, Neurodegeneration, Amsterdam 1081 HV, The Netherlands; Alzheimer Center Amsterdam UMC, Department of Neurology, Amsterdam 1081 HV, The Netherlands; Amsterdam Neuroscience, Brain Imaging, Amsterdam 1081 HV, The Netherlands; Department of Anatomy and Neurosciences, Amsterdam UMC, Amsterdam 1081 HV, The Netherlands; Amsterdam Neuroscience, Neurodegeneration, Amsterdam 1081 HV, The Netherlands; Amsterdam Neuroscience, Brain Imaging, Amsterdam 1081 HV, The Netherlands; Department of Anatomy and Neurosciences, Amsterdam UMC, Amsterdam 1081 HV, The Netherlands; Amsterdam Neuroscience, Neurodegeneration, Amsterdam 1081 HV, The Netherlands; Amsterdam Neuroscience, Brain Imaging, Amsterdam 1081 HV, The Netherlands; Department of Anatomy and Neurosciences, Amsterdam UMC, Amsterdam 1081 HV, The Netherlands; Amsterdam Neuroscience, Neurodegeneration, Amsterdam 1081 HV, The Netherlands; Amsterdam Neuroscience, Brain Imaging, Amsterdam 1081 HV, The Netherlands; Department of Radiology and Nuclear Medicine, Amsterdam UMC, Amsterdam 1081 HV, The Netherlands; Queen Square Institute of Neurology and Centre for Medical Image Computing, University College London, London WC1N 3BG, UK; Amsterdam Neuroscience, Neurodegeneration, Amsterdam 1081 HV, The Netherlands; Alzheimer Center Amsterdam UMC, Department of Neurology, Amsterdam 1081 HV, The Netherlands; Amsterdam Neuroscience, Brain Imaging, Amsterdam 1081 HV, The Netherlands; Department of Anatomy and Neurosciences, Amsterdam UMC, Amsterdam 1081 HV, The Netherlands; Amsterdam Neuroscience, Neurodegeneration, Amsterdam 1081 HV, The Netherlands; Amsterdam Neuroscience, Brain Imaging, Amsterdam 1081 HV, The Netherlands; Department of Pathology, Amsterdam UMC, Amsterdam 1081 HV, The Netherlands; Amsterdam Neuroscience, Neurodegeneration, Amsterdam 1081 HV, The Netherlands; Alzheimer Center Amsterdam UMC, Department of Neurology, Amsterdam 1081 HV, The Netherlands; Department of Pathology, Amsterdam UMC, Amsterdam 1081 HV, The Netherlands; Department of Anatomy and Neurosciences, Amsterdam UMC, Amsterdam 1081 HV, The Netherlands; Amsterdam Neuroscience, Neurodegeneration, Amsterdam 1081 HV, The Netherlands; Amsterdam Neuroscience, Brain Imaging, Amsterdam 1081 HV, The Netherlands

**Keywords:** Alzheimer’s disease, MRI, TDP-43, amygdala, neuropathology, Lewy body disease

## Abstract

Limbic-predominant age-related TDP-43 encephalopathy neuropathological change (LATE-NC) refers to the aberrant accumulation of TDP-43 in the brains of ageing individuals either in isolation or in combination with neurodegenerative disease. LATE-NC is most commonly found in the amygdala and hippocampus and is associated with progressive amnestic decline in individuals with a neurodegenerative disease. Since LATE-NC can only be diagnosed post-mortem, there is a need for pathology-validated neuroimaging biomarkers for LATE-NC. In the current study we assessed MRI-measured amygdalar and hippocampal volume in brain donors with Alzheimer's disease or Lewy body diseases with and without co-occurring LATE-NC pathology.

Post-mortem *in situ* 3D-T1 3T-MRI data were collected for 51 cases (27 Alzheimer's disease and 24 Lewy body disease) of whom 17 had post-mortem confirmed LATE-NC and 34 were non-LATE-NC (matched on age, sex and neurodegenerative disease). Amygdalar and hippocampal volumes were calculated using FreeSurfer. Within-subject amygdalar and hippocampal tissue sections were immunostained for TDP-43 (pTDP-43), phosphorylated tau (AT8), amyloid-β (4G8) and α-synuclein (pSer129). Positive cell density (TDP-43 and α-synuclein) and area percentage immunoreactivity (p-tau and amyloid-β) outcome measures were quantified using QuPath. Group differences between LATE-NC and non-LATE-NC donors were assessed with univariate analyses and correlations were assessed with linear regression models, all adjusting for intracranial volume and post-mortem delay and if applicable for primary pathology.

Brain donors with LATE-NC showed significantly lower amygdalar (−26%, *P* = 0.014) and hippocampal (−19%, *P* = 0.003) volumes than non-LATE-NC brain donors, even when correcting for regional phosphorylated tau, amyloid-β and α-synuclein burden. These group differences remained significant in the Alzheimer's disease group (amygdala −24%, *P* = 0.028; hippocampus −21%, *P* = 0.002), but in the Lewy body diseases group only the amygdala was smaller in LATE-NC donors compared with non-LATE-NC donors (18%, *P* = 0.030). These results suggest that severity of TDP-43 burden plays a role in amygdala and hippocampus atrophy on MRI, even when correcting for effects of primary pathology. This study proposes that exceptionally low amygdalar and hippocampal volumes could indicate LATE-NC and that this may serve as a potential biomarker for *in vivo* studies.

## Teaser

Using post-mortem *in situ* MRI and immunohistochemistry, Wesseling *et al.* show that brain donors with LATE-NC have lower amygdalar and hippocampal volumes than non-LATE-NC donors, even when correcting for regional phosphorylated tau, amyloid-beta, and alpha-synuclein burden.

## Introduction

Limbic-predominant age-related TDP-43 encephalopathy neuropathological change (LATE-NC) is a recently proposed neuropathological entity that involves the aggregation of TDP-43 protein in the amygdala, hippocampus and cortical regions.^1^ Recent autopsy-based studies have found the prevalence of LATE-NC to be 20%–55% in individuals older than 80 years.^2-4^ In Alzheimer's disease (AD) patients, the prevalence of comorbid LATE-NC pathology has been reported to be between 20% and 60%,^1,5,6^ with a lower prevalence of around 7%–10% in early-onset AD (EOAD, age-at-onset <65 years).^7^ Fewer studies have investigated the prevalence of LATE-NC pathology in Lewy body diseases (LBD), but LATE-NC seems to be more common in Parkinson's disease dementia (PDD; 19%) and dementia with Lewy bodies (DLB; 30%–45%) compared with individuals with Parkinson's disease (PD) without dementia (7.2%).^8,9^ The prevalence of LATE-NC in LBD might be higher in those with limbic- or amygdala-predominant LBD as opposed to neocortical-predominant LBD.^10^

LATE-NC is associated with impaired cognition in two ways: LATE is a clinical syndrome and a cause of cognitive decline independent of other neurodegenerative diseases^1,11^ but LATE-NC is also prevalent as a co-pathology in patients with neurodegenerative diseases.^12^ In AD, those with LATE-NC pathology exhibit faster cognitive decline compared with AD patients without LATE-NC.^13,14^

Currently, there is no standardized neuroimaging-based assessment for LATE-NC during life, and it remains difficult to assess the extent to which LATE-NC pathology plays a role in the cognitive profile of individuals during their lifetime. Several studies explored *in vivo* magnetic resonance imaging (MRI) with subsequent autopsy validation as a neuroimaging marker for LATE-NC in AD. They found more pronounced hippocampal atrophy in LATE-NC compared with non-LATE-NC AD donors.^15-18^ More specifically, MRI volume and TDP-43 pathology were negatively associated in the anterior hippocampus^18^ and cornu ammonis (CA) 1 region, indicating hippocampal (subfield) volume as a potential marker for LATE-NC.^15^ Intervals of several years can limit the interpretation of post-mortem immunohistochemistry measures to *in vivo* MRI data, because disease progression may obscure the role of LATE-NC pathology in the neurodegenerative process. Post-mortem MRI can overcome this challenge, as it has the unique advantage of allowing a direct comparison between imaging and pathology at the same moment in time. Several studies have used *ex vivo* post-mortem imaging of formalin-fixed brain tissue to investigate neuropathological correlates of amygdalar and hippocampal atrophy,^19-21^ and show that both p-tau and TDP-43 severity contribute to amygdalar and hippocampal volume loss.^18,21^ Moreover they show that TDP-43 burden associates with amygdalar and hippocampal shape distortion,^19,21^ suggesting that amygdalar and hippocampal subfields might be differently affected by LATE-NC pathology. Nevertheless, volumetric MRI studies performed on fixed brain tissue impair comparability to *in vivo* MRI, as brain tissue can be distorted during the fixation process. Post-mortem *in situ* (brain still in cranium) MRI could mitigate this limitation.^22^

The aim of the current study was to investigate the association between LATE-NC pathology and post-mortem *in situ* MRI volume in a cohort of brain donors with AD and LBD. Furthermore, we aimed to investigate whether differences in amygdalar and hippocampal volume between LATE-NC and non-LATE-NC AD donors could already be identified on preceding *in vivo* MRI. Lasty, we aimed to explore the relative contribution of TDP-43, p-tau, amyloid-β and α-synuclein pathology on post-mortem MRI volumes. Results of this study could provide insights towards amygdalar and hippocampal MRI volumes as neuroimaging biomarkers for LATE-NC.

## Materials and methods

### Donor inclusion

**Figure 1:**
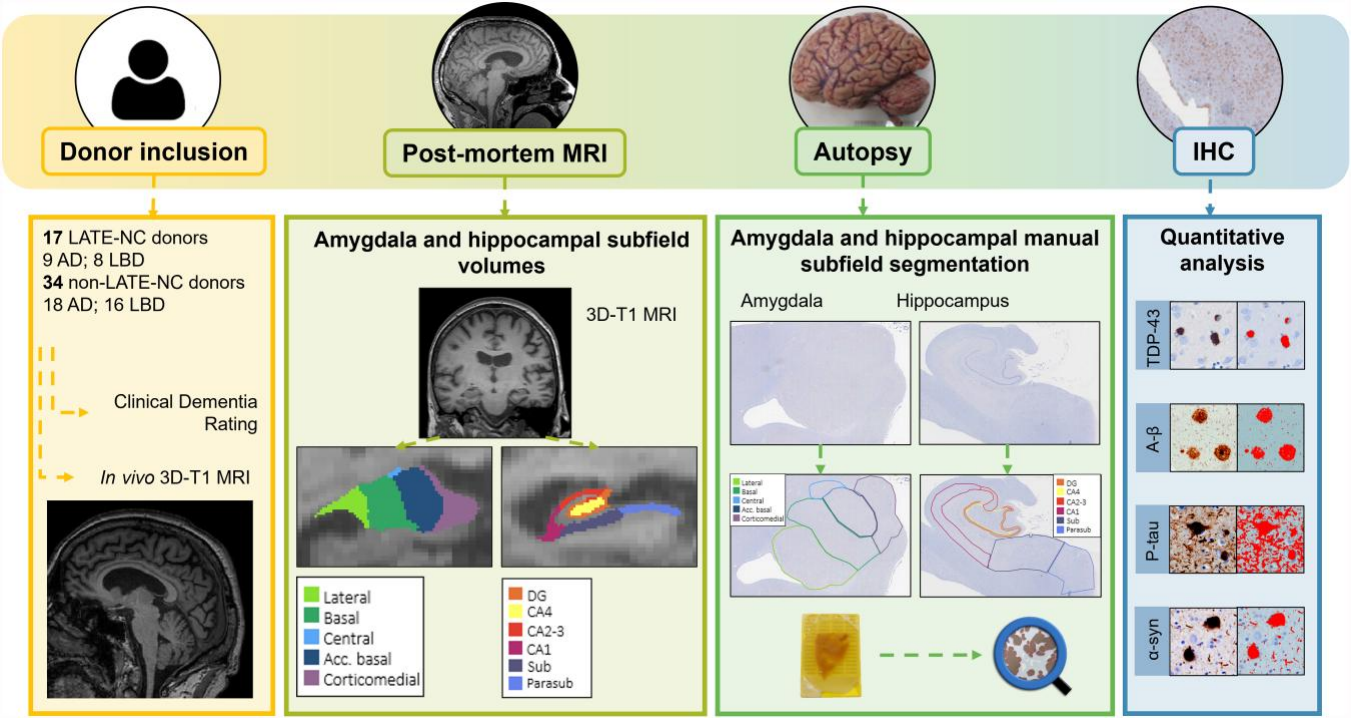
**Post-mortem MRI-pathology pipeline.** For 51 donors, post-mortem 3 T *in situ* MRI was obtained, and amygdala and hippocampal subfield volumes were calculated with FreeSurfer^23,24^ from the 3D-T1 image (yellow box). Autopsy was performed after MRI acquisition, and amygdala and hippocampal tissue was processed for immunohistochemistry against TDP-43, Aβ, p-tau and α-syn (green and blue boxes). Regions of interest were drawn for the hippocampal subfields^25^ and amygdala subnuclei,^26^ to include the same regions as segmented on MRI (green box). Final quantitative outcome measures were obtained using object and pixel classifiers in Qupath (blue box).^27^ AD = Alzheimer's disease; LBD = Lewy body disease; Aβ = amyloid-beta; α-syn = α-synuclein; IHC = immunohistochemistry.

For an overview of our workflow, see Fig. 1. A total of 51 brain donors with clinically diagnosed and pathologically confirmed AD or LBD with available post-mortem *in situ* MRI were included in this study. Of these, 45 donors were included in collaboration with the Netherlands Brain Bank (http://brainbank.nl) and 6 donors were included in collaboration with the Normal Aging Brain Collection Amsterdam (www.nabca.eu). Neuropathological diagnosis was established by an expert neuropathologist (A.M.R.) according to the international guidelines of the BrainNet Europe II consortium (BNE).^28^ Assessment of hippocampal sclerosis was performed on haematoxylin and eosin-stained hippocampal sections (Supplementary Fig. 1). Neuropathological assessment of LATE-NC pathology was done according to the guidelines by Nelson *et al*.^1^ According to these guidelines, LATE-NC stage 1 includes TDP-43 positivity in either the amygdalar or (para)hippocampal regions, spreading to both regions in stage 2 and to the middle frontal gyrus in stage 3.^1,29^

Donors with a LATE-NC stage of 1, 2 or 3 (*n* = 9 AD, *n* = 8 LBD) were retrospectively selected from existing cohorts of donors with AD or LBD. A group of donors without LATE-NC pathology (*n* = 18 AD, *n* = 16 LBD) from the same cohorts was matched based on age, sex and neuropathological staging within disease group (Braak neurofibrillary tangle (NFT) stage,^30^ Thal phase^31^ and Braak Lewy Body stage).^32^ If available, Clinical Dementia Rating (CDR) scores for all donors were obtained from the last clinical assessment or retrospectively by a general practitioner as a measure of cognitive decline. The donors had previously provided consent for the use of their brain tissue for research purposes.

### MRI acquisition: *in situ* and *in vivo*

All donors underwent post-mortem 3 T *in situ* MRI according to a previously described pipeline.^33^ Scans were acquired on a 3 T scanner (Sigma-MRI750, General Electric Medical Systems) with an eight-channel phased-array head coil. T1-weighted images were acquired using a sagittal 3D-T1-weighted fast spoiled gradient echo (GRE) sequence [repetition time (TR) = 7 ms; echo time (TE) = 3 ms; inversion time (TI) = 450 ms; flip angle = 15°; slice thickness = 1 mm; in-plane resolution = 1.0 × 1.0 mm^2^]. A sagittal 3D fluid attenuation inversion recovery (FLAIR) sequence was acquired with the following parameters: TR = 8000 ms; TE = 130 ms; TI = 2000–2250 ms; slice thickness = 1.2 mm; in-plane resolution = 1.11 × 1.11 mm^2^. The inversion time of the post-mortem FLAIR sequence was optimized for each individual case to account for differences in CSF suppression due to body temperature.

Furthermore, 19 of 24 AD cases included in this study had ante-mortem *in vivo* 3 T MRI scans available, retrospectively included from the Amsterdam Dementia Cohort^34^ using different scanners. For their T1-weighted images, the TR varied between 7.8 and 7.9 ms, and TE between 2.9 and 5.2 ms (Supplementary Table 1) with voxel size fixed at 1 mm^3^ isotropic. If more than one *in vivo* MRI was available, the most recent one was used for our analysis. Time between most recent *in vivo* MRI and death was between 4 days and 6 years with a mean of 3.3 years [standard deviation (SD) 2.0 years].

### MRI brain volume assessment

All post-mortem 3D-T1 images were lesion-filled based on the FLAIR images as previously described,^35^ to reduce the effect of vascular burden on their tissue type segmentation. Parcellation of brain areas was performed with Freesurfer (Freesurfer), version 7.0,^36^ using the Dekisan–Killarney atlas,^37^ from which intracranial volume, amygdalar and hippocampal volumes from both hemispheres were extracted. Using the Freesurfer atlas by Saygin *et al*.,^23^ the following amygdalar subfields were identified: lateral nucleus, basal nucleus, accessory basal nucleus, corticomedial nuclei (including the cortico-amygdaloid transition area, cortical and medial nuclei) and the central nucleus. Furthermore, the dentate gyrus, CA4 region, CA3 (including CA2) region, CA1 region, subiculum and parasubiculum (including the presubiculum) were identified using the Hippocampal Subfield protocol with no head/body division (suffix FS60) for the hippocampus.^24^ Additionally, visual MRI scores (Global cortical atrophy, parietal cortical atrophy, medial temporal lobe atrophy^38^ and Fazekas score^39^) were determined by a clinical radiologist (F.B.).

### Tissue sampling

Autopsy was performed directly after *in situ* MRI acquisition, which resulted in a post-mortem delay of less than 13 h for all subjects (mean post-mortem delay 7 h and 35 min). Tissue blocks from the amygdala and hippocampus were collected from the right hemisphere after 4 weeks fixation. The hippocampal blocks were cut from the middle of the hippocampus to ensure inclusion of all subfields. The amygdalar blocks were cut to include the anterior part of the entorhinal cortex. All tissue blocks were subsequently paraffin embedded for immunohistochemistry.

### Immunohistochemistry

Sections of 6 µm were cut from the amygdalar and hippocampal blocks and mounted on superfrost+ glass slides (Thermo Fisher Scientific). Amygdalar and hippocampal tissue from all donors were stained for phosphorylated tau (p-tau, AT8, Thermo Fisher Scientific, 1:800 dilution), amyloid-β (4G8, Biolegend, 1:4000 dilution), phosphorylated α-synuclein (pSer-129, Abcam, 1:8000 dilution) and phosphorylated TDP-43 (pTDP-43, Cosmo Bio, 1:8000 dilution). All deparaffinized tissue sections were immersed in 10 mM citrate buffer (pH 6.0) for TDP-43, 4G8 and AT8 and in 10 mM Tris-EDTA buffer (pH 9.0) for pSer-129 and heated to 120°C in a steam cooker for antigen retrieval. Sections were blocked for 30 min for endogenous peroxidase (0.3%) in PBS for TDP-43 (PBS; pH 7.4) and in Tris-buffered saline for pSer-129, 4G8 and AT8 (TBS; pH 7.4). Primary antibodies were diluted in PBS for TDP-43 and TBS for pSer-129, AT8 and 4G8 with 1% normal goat serum (ImmunoLogic) and 0.5% Triton and incubated overnight at 4°C. Primary antibodies were detected using EnVision (Dako) and visualized with 3.3′-diaminobenzidine (DAB, Dako) with Imidazole (50 mg DAB, 350 mg Imidazole and 30 µl of H_2_O_2_ per 100 ml of Tris-HCl 30 mM, pH 7.6). PBS (pTDP-43) or TBS (pSer-129; 4G8; AT8) was used in between steps to wash the sections. Finally, sections were counter-stained with haematoxylin, dehydrated and mounted with Entellan (Merck).

### Image analysis

A whole-slide scanner (Olympus VS200; Evident; 20× objective) was used to visualize the sections which were subsequently quantified using QuPath version 3.2.0.^27^ Hippocampal regions of interest were drawn to include the dentate gyrus, CA1-4 and (para)subiculum based on cytoarchitectural boundaries as previously described by Adler et al.^25^ The whole hippocampus was defined as the sum of these regions. The amygdala and its subnuclei were first segmented using Nissl staining, after which the different subnuclei were identified and segmented on neuropathological stainings. The amygdala was segmented into the lateral, basal, accessory basal, central (including the central lateral and the central medial nuclei) and cortico-medial nuclei (including the peri-amygdaloid cortex, the cortical nuclei and the medial nuclei) according to Schumann et al.^26^

For TDP-43 and α-synuclein, positive cells were counted using an object classifier, resulting in an outcome measure representing the Lewy body/TDP-43 positive cell inclusion density in a region. For p-tau and amyloid-β, immunoreactivity of DAB staining was quantified using previously published pixel classifiers,^40,41^ which resulted in an outcome measure representing area % immunoreactivity (Supplementary Fig. 2).

### Statistics

Statistical analysis was performed using IBM SPSS 28.0 for Windows (SPSS, Inc., Chicago, USA) and R studio 4.4.3 (Boston, USA, http://www.posit.co). All variables were tested for normality by visual inspection. MRI volume and p-tau load were normally distributed, but α-synuclein inclusion density, TDP-43 inclusion density and amyloid-β load were not. These were log transformed, after which the transformed amyloid-β load and α-synuclein density were normally distributed. The log transformed TDP-43 positive cell count was still not normally distributed, and thus a Spearman rank test was used to investigate correlations with this variable. Demographics between groups were compared using the Mann–Whitney U-test for continuous data and Fisher exact test for categorical data.

An analysis of covariance (ANCOVA) was used to calculate group differences in MRI volume between LATE-NC and non-LATE-NC donors, both in the whole cohort and within AD and LBD separately. For analyses using post-mortem MRI volume data, post-mortem delay and intracranial volume were included as covariates, while for the *in vivo* MRI analysis the intracranial volume (ICV) and time between *in vivo* and post-mortem MRI scans (in years) were controlled for. For analyses where primary pathology was corrected for, amyloid-β, p-tau and/or α-synuclein were included as covariates in the analysis. Since LATE-NC and non-LATE-NC donors were matched on sex and age and these variables did not differ between groups, these variables were not added as covariates in these analyses. Group differences in pathological load of p-tau, amyloid-β, α-synuclein and TDP-43 were calculated using *t*-tests to account for non-equal variance between groups. Analyses of the different amygdalar and hippocampal subfields were false discovery rate (FDR) corrected for multiple comparisons. For estimation of the receiver operating characteristic (ROC) curve, R version 4.4.1 (2024-06-14 ucrt) and the pROC^42^ package were used.

Correlations between amyloid-β, α-synuclein and p-tau were investigated using Pearson correlations, while correlations between TDP-43 and amyloid-β, α-synuclein or p-tau were assessed using Spearman rank tests. Correlations between pathologies were only performed in donors with pathology in this region. For the amygdala this was defined as LATE-NC ≥1, Braak LB ≥4, Braak NFT ≥4 and Thal ≥3. For the hippocampus this was defined as LATE-NC ≥2, Braak LB ≥3, Braak NFT ≥1 and Thal ≥2. Correlations between different pathological measures were FDR corrected for multiple comparisons.

To investigate the combined effect of TDP-43, p-tau, α-synuclein and amyloid-β on amygdalar and hippocampal volume, and to identify which pathological hallmark(s) was/were the strongest driver(s) of MRI volume loss, linear regression analysis with backward selection was performed. This model included MRI volume as a dependent variable and included the four pathological markers in a backward model as well as intracranial volume as a separate variable.

## Results

### Donor characteristics

Demographic, clinical, radiological and pathological data from donors are summarized in Table 1. Clinical diagnosis, age at death, sex and Fazekas score^39^ did not differ between LATE-NC and non-LATE-NC groups, whereas LATE-NC donors had a shorter average disease duration (*P* = 0.047), as well as higher CDR scores^43^ (*P* = 0.020) and medial temporal lobe atrophy (MTA)^38^ scores (*P* = 0.014). LATE-NC donors did not differ in post-mortem delay (PMD), *APOE4* genotype, presence of atherosclerosis, Thal phase,^31^ Braak LB^32^ or Braak NFT^30^ stage from non-LATE-NC donors. LATE-NC donors also had a higher incidence of hippocampal sclerosis (*P* = 0.001). For a summary of individual data, see Supplementary Table 2.

**Table 1:** Demographic, clinical, radiological, and pathological data of included donors

	LATE (*n* = 17)	Non-LATE (*n* = 34)	*P*-value
Clinical diagnosis, AD/PD/PDD/DLB	9/2/2/4	18/12/4/0	0.583
Sex, female/male (% female)	8/9 (47.1)	14/20 (41.2)	0.458
Age at death, years, mean (range)	88 (61–94)	75 (53–93)	0.056
PMD, min, mean (range)	440 (249–640)	463 (210–715)	0.504
Disease duration, years, mean (range)	7 (1–16)	11 (1–23)	0.047^*^
CDR score, NA/0/0,5/1/2/3	8/0/0/1/1/7	13/0/4/7/3/7	0.020^*^
MTA score, NA/0/1/2/3/4	1/0/3/3/3/7	3/0/13/10/6/2	0.014^*^
Fazekas score, NA/0/1/2/3	1/2/3/3/8	2/5/10/10/7	0.103
*In vivo* MRI available, *n*	5	12	NA
LATE stage 1/2/3	0/13/4	NA	NA
Hippocampal sclerosis, *n* (%)	6 (35)	1 (3)	0.001^**^
Atherosclerosis, *n* (%)	15 (88)	24 (71)	0.459
ARTAG, *n* (%)	4 (24)	12 (50)	0.608
Thal Amyloid-β, 0/1/2/3/4/5	1/3/0/1/2/10	1/6/3/6/1/17	0.594
Braak NfT, 0/1/2/3/4/5/6	0/0/0/4/5/1/7	0/3/9/3/4/8/7	0.078
Braak Lewy body, 0/1/2/3/4/5/6	7/0/1/0/0/1/8	17/0/0/1/2/2/12	0.610
APOε^a^, %ε4-allele (*n*)	77.8 (7)	66.7 (12)	0.450

AD, Alzheimer's disease; ARTAG, age-related tau astrogliopathy; CDR, clinical disease rating; DLB, dementia with Lewy bodies; MTA, medial temporal lobe atrophy; NA, not available; NFT, neurofibrillary tangle; PD, Parkinson's disease; PDD, Parkinson's dementia; PMD, post-mortem delay.

^a^Only available for AD donors.

^*^ *P* < 0.05.

^**^ *P* < 0.01.

### LATE-NC donors have lower amygdalar and hippocampal MRI volumes

**Figure 2:**
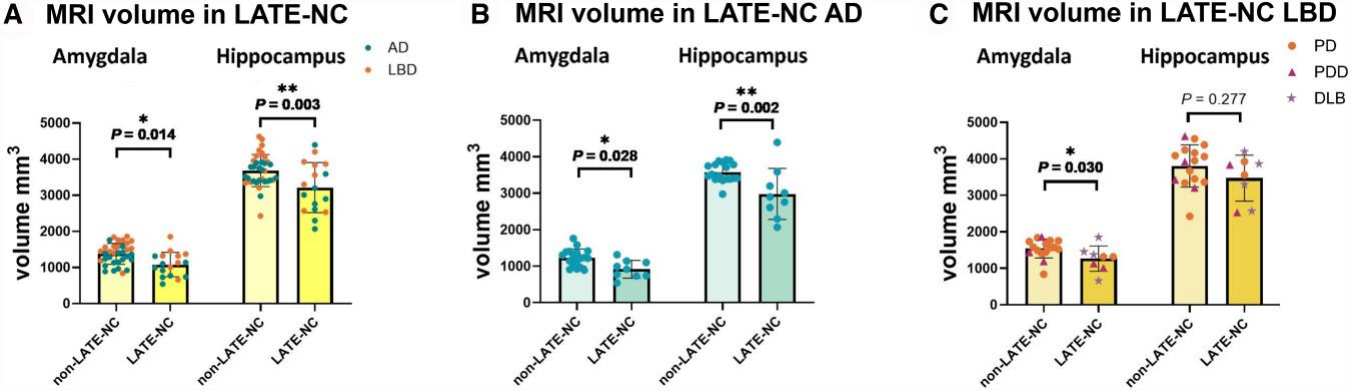
**Differences in MRI volume between LATE-NC and non-LATE-NC donors.** (**A**) Difference in amygdala and hippocampal volume (in mm^3^) between limbic-predominant age-related TDP-43 encephalopathy neuropathological change (LATE-NC) and non-LATE-NC donors. (**B**) Difference in amygdala and hippocampal volume between LATE-NC Alzheimer's disease (AD) and non-LATE-NC AD donors. (**C**) Difference in amygdala and hippocampal volume between LATE-NC Lewy Body disease (LBD) and non-LATE-NC LBD donors. *P < 0.05; **P < 0.01. DLB = dementia with Lewy bodies; PD = Parkinson's disease; PDD = Parkinson's disease dementia.

Without correction for primary pathology, brain donors with LATE-NC had a 22% lower right hemisphere (ipsilateral to pathological data) amygdalar (*P* = 0.001) and 13% lower hippocampal (*P* = 0.009) volume compared with non-LATE-NC donors. Furthermore, a significant reduction of 15% in amygdalar (*P* = 0.035) but not hippocampal volume (*P* = 0.842) was found in the left hemisphere. When correcting for p-tau, amyloid-β and α-synuclein, the difference in volume between LATE-NC and non-LATE-NC donors increased for the amygdala (26% volume reduction, *P* = 0.014) and hippocampus (19% volume reduction, *P* = 0.003) of the right hemisphere (Fig. 2A), but was not significant for the amygdala and hippocampus of the left hemisphere.

**Figure 3:**
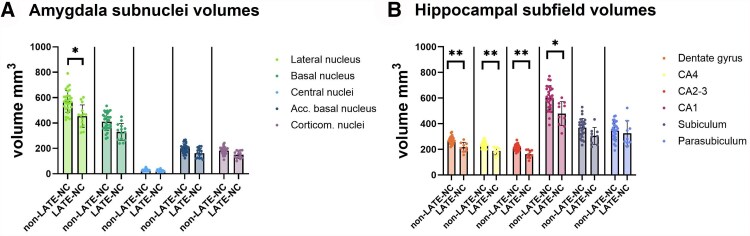
**Amygdala and hippocampal subregion volume differences in amygdala subnuclei (A) and hippocampal (B) subfield volumes in limbic-predominant age-related TDP-43 encephalopathy neuropathological change (LATE-NC) and non-LATE-NC donors.** *P < 0.05; **P < 0.01.

Within the right hemisphere, when investigating different amygdala subnuclei, only the lateral nucleus (−20%, *P* = 0.014) was significantly smaller in LATE-NC donors compared with non-LATE-NC donors (Fig. 3A). In the hippocampus, the dentate gyrus (−20%, *P* = 0.002), CA1 (−20%, *P* = 0.027), CA2-3 (−24%, *P* = 0.002) and CA4 (−22%, *P* = 0.002) were significantly smaller in LATE-NC compared with non-LATE-NC donors (Fig. 3B).

**Figure 4:**
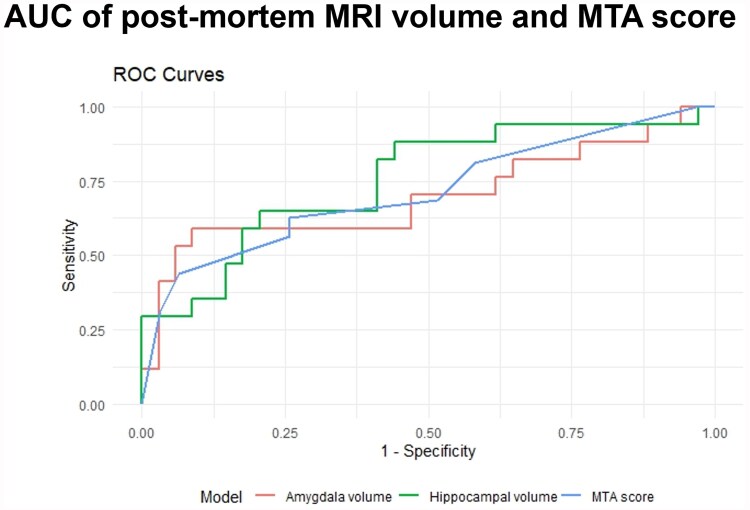
**Area under the curve for medial temporal lobe atrophy score and amygdalar and hippocampal volumes.** The area under the curve (AUC) distinguishing limbic-predominant age-related TDP-43 encephalopathy neuropathological change(LATE-NC) from non-LATE-NC donors is similar for medial temporal lobe atrophy (MTA) score (0.716), (intracranial volume corrected) amygdalar (0.777) and hippocampal (0.715) volumes. ROC = receiver operator characteristic.

To investigate whether MRI-measured amygdala and hippocampal volume are valuable predictors of LATE-NC we created a ROC curve to visualize the area under the curve (AUC) for MTA score, amygdalar and hippocampal volume (corrected for ICV). The AUC of amygdalar (0.777) and hippocampal (0.715) volume was similar compared with that of MTA score (0.715) (Fig. 4).

### MRI atrophy patterns differ between LATE-NC AD and LATE-NC LBD donors

When investigating LATE-NC AD and LBD donors separately, LATE-NC AD donors showed a similar pattern with smaller amygdalar (−26%, *P* = 0.009) and hippocampal (−17%, *P* = 0.007) volumes in the right hemisphere but not in the left hemisphere (amygdala: *P* = 0.146; hippocampus: *P* = 0.457) compared with non-LATE-NC AD donors. These group differences remained when controlling for p-tau and amyloid-β (amygdala: 24%, *P* = 0.028; hippocampus: 21%, *P* = 0.002) (Fig. 2B). LATE-NC LBD donors only had smaller amygdalar volumes (−19%, *P* = 0.045) but no hippocampal (*P* = 0.336) or left hemisphere (amygdala: *P* = 0.158; hippocampus: *P* = 0.715) volume difference compared with non-LATE-NC LBD donors. These results remained the same when controlling for α-synuclein (amygdala: −18%, *P* = 0.030; hippocampus: *P* = 0.277) (Fig. 2C).

### Post-mortem hippocampal volume reflects *in vivo* volume

**Figure 5:**
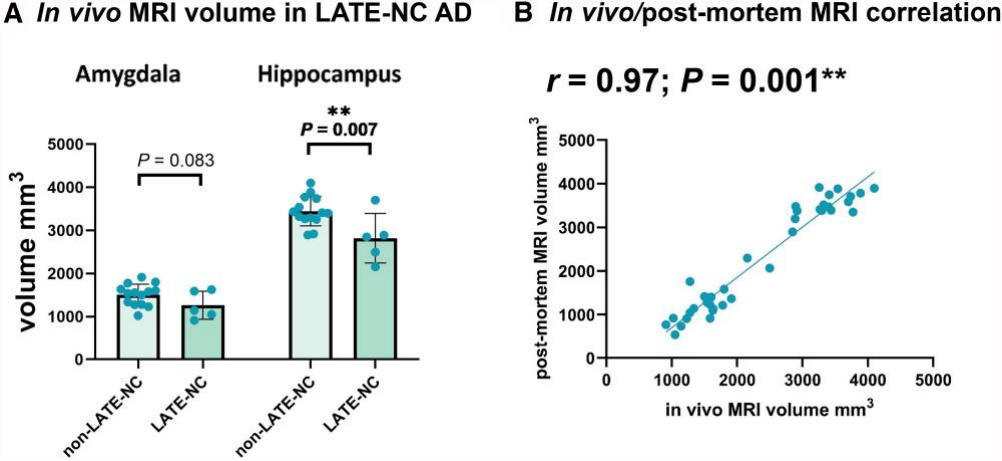
***In vivo* MRI volumes.** (**A**) Differences in *in vivo* amygdala and hippocampal volumes between limbic-predominant age-related TDP-43 encephalopathy neuropathological change (LATE-NC) and non-LATE-NC Alzeimer's disease (AD) donors. (**B**) Correlation between post-mortem and *in vivo* MRI, corrected for amount of time between the two scans.

*In vivo* MRI was available for 5 LATE-NC and 12 non-LATE-NC donors. When comparing *in vivo* MRI volumes between LATE-NC and non-LATE-NC AD donors, we found that hippocampal (18%, *P* = 0.007), but not amygdalar (*P* = 0.083) volumes were significantly lower in LATE-NC donors (Fig. 5A). Figure 5B shows the correlation between *in vivo* and post-mortem MRI volume, corrected for time between the two MRI scans (*r* = 0.97; *P* = 0.001).

### Neuropathological burden in LATE-NC and non-LATE-NC

**Figure 6:**
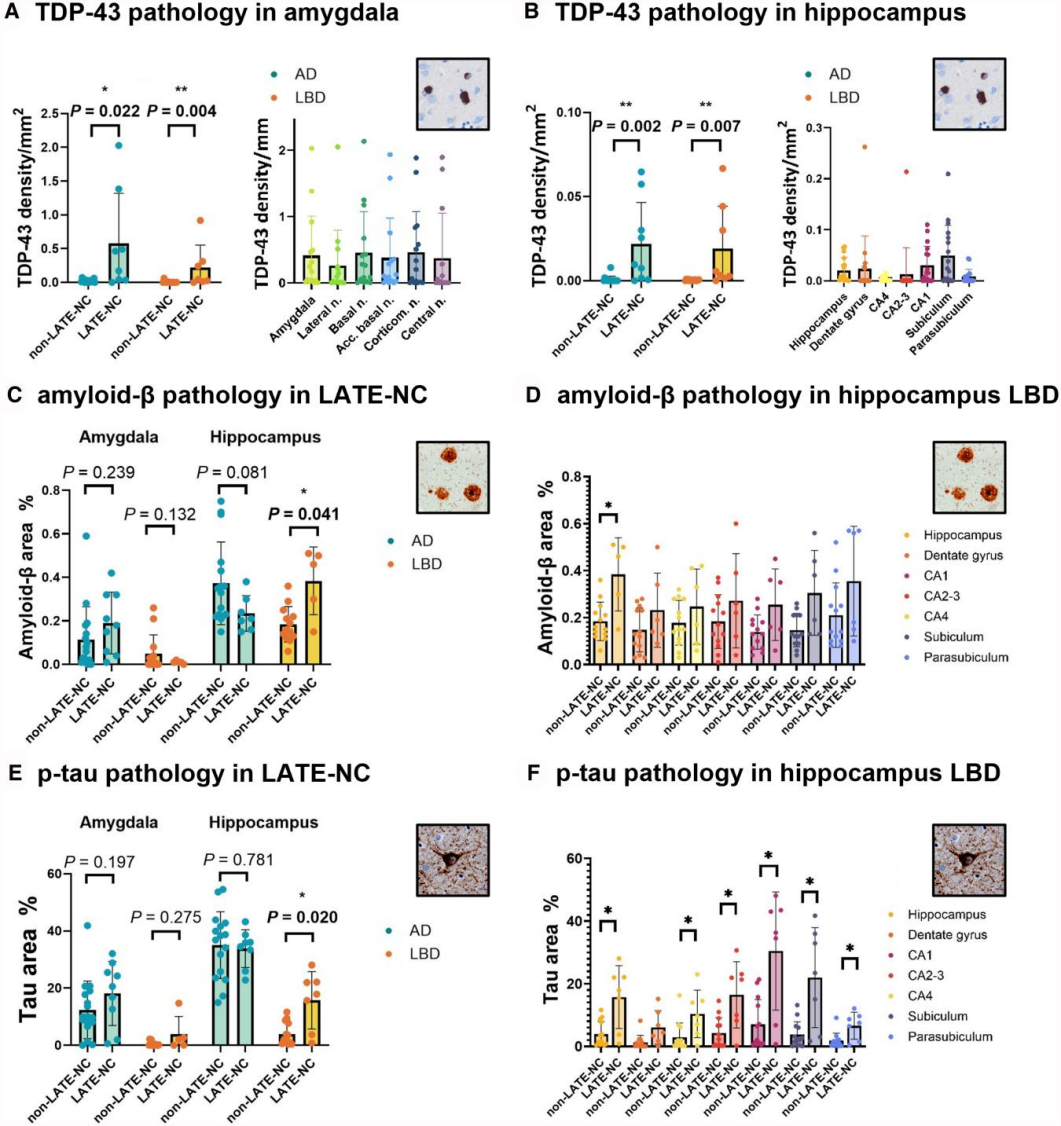
**Differences in pathological load between limbic-predominant age-related TDP-43 encephalopathy neuropathological change (LATE-NC) and non-LATE-NC donors.** Difference in TDP-43 density (**A** and **B**) amyloid-β load (**C**and **D**) and p-tau load (**E** and **F**) in LATE-NC and non-LATE-NC donors. *P < 0.05; **P < 0.01. AD = Alzheimer's disease; LBD = Lewy body disease; p-tau = phosphorylated tau.

By definition, LATE-NC donors had significantly higher TDP-43 positive cell density in the amygdala (*P* = 0.023) and hippocampus (*P* = 0.004) compared with non-LATE-NC donors (Fig. 6A and B). When investigating different amygdalar and hippocampal subregions, we found significantly higher TDP-43 pathology in all amygdala subnuclei, as well as in the CA1, CA4, subiculum and parasubiculum of the hippocampus. LATE-NC AD donors did not differ from non-LATE-NC AD donors on pathological load of p-tau, amyloid-β or α-synuclein density. However, LATE-NC LBD donors had significantly higher amyloid-β (*P* = 0.041) and p-tau load (*P* = 0.020) in the hippocampus compared with non-LATE-NC LBD donors (Fig. 6C and E). More specifically, LATE-NC LBD donors had higher p-tau load in all individual hippocampal subfields (Fig. 6F). None of the hippocampal subregions showed significantly higher amyloid-β load in LATE-NC LBD donors compared with non-LATE-NC LBD donors (Fig. 6D).

For a detailed overview of observed correlations between pathologies, please refer to Supplementary Table 3. P-tau and amyloid-β load were positively correlated in the whole hippocampus (*r* = 0.51; *P* = 0.003) and all subfields separately, as well as in the accessory basal nucleus of the amygdala (*r* = 0.54; *P* = 0.030). No significant correlations between other pathologies including TDP-43 were reported after FDR correcting for multiple comparisons.

### TDP-43 predicts amygdalar volume in LATE-NC donors

To explore which pathological aggregate(s) (TDP-43, p-tau, α-synuclein and amyloid-β), best explained variance in amygdalar and hippocampal volume, we conducted an exploratory analysis using a linear regression model of our entire cohort. The final model predicting amygdalar volume included TDP-43 (*β* = −0.24, *P* = 0.091), p-tau (*β* = −0.33, *P* = 0.020) and intracranial volume (*β* = 0.45, *P* = 0.001), and had a total adjusted *R^2^* of 0.401. The final model predicting hippocampal volume included TDP-43 (*β* = −0.38, *P* = 0.002), p-tau (*β* = −0.26, *P* = 0.030) and intracranial volume (*β* = 0.42, *P* = 0.001), and had a total adjusted *R^2^* of 0.515. Supplementary Fig. 4 shows the volume of donors with different combinations of neuropathologies.

We also performed the same prediction model as above in the LATE-NC group only. Among the four pathological aggregates, TDP-43 was the strongest predictor of amygdalar volume within LATE-NC donors. The final model included TDP-43 (*β* = −0.67, *P* = 0.013) and intracranial volume (*β* = 0.98, *P* = 0.002) as significant predictors and had a total adjusted *R^2^* of 0.631. The model that best predicted hippocampal volume included only intracranial volume (*β* = 0.78, *P* = 0.002) and none of the pathological aggregates.

## Discussion

Using a post-mortem *in situ* MRI and concurrent immunohistochemistry approach, we found that LATE-NC donors had lower amygdalar and hippocampal volumes compared with non-LATE-NC donors, even when accounting for the contribution of primary amyloid-β, p-tau and α-synuclein pathologies. Furthermore, we found that TDP-43 cell density predicted volume loss in the amygdala of LATE-NC donors. These findings suggest that TDP-43 pathology in LATE-NC plays a role in amygdalar volume loss, independently of primary pathology.

In line with earlier literature,^5^ LATE-NC donors had higher CDR scores compared with non-LATE-NC donors, indicative of more cognitive impairment. This finding, in combination with the shorter disease duration in LATE-NC compared with non-LATE-NC donors, suggests that donors with LATE-NC have a faster cognitive decline. Early clinical recognition of LATE-NC co-pathology is therefore important for an accurate clinical prognosis. Interestingly, 2 of 17 LATE-NC donors had a disease onset before 65 years, which suggests that it is important to consider LATE-NC not just as a pathology in older individuals.^1,44^

In LATE-NC donors, our study found a decrease in post-mortem amygdalar volume between 22% and 26% as well as a decrease between 15% and 19% for hippocampal volume, depending on hemisphere and disease group. This is in line with Bejanin et al. (2019) reporting between 5% and 15% *in vivo* amygdalar and hippocampal volume loss in neuropathological confirmed LATE-NC compared with non-LATE-NC individuals.^16^ Several other studies reporting amygdalar and/or hippocampal volume loss in LATE-NC donors, do not specify the exact difference in MRI volumes.^17,19^ Interestingly, the volume loss in LATE-NC compared with non-LATE-NC donors was greater (26% amygdala and 20% hippocampus) after controlling for primary pathology, suggesting that the amount of atrophy that can be contributed to LATE-NC pathology might be greater than previously thought.

Lower volumes of the medial temporal lobe in LATE-NC donors were also reflected by lower post-mortem MTA scores^38^ in LATE-NC donors compared with non-LATE-NC donors. Furthermore, MTA seemed to be equal to hippocampal and amygdalar volume in distinguishing LATE-NC from non-LATE-NC. The MTA score has previously been reported to be a sensitive marker for hippocampal atrophy in AD,^45^ but according to our study it might also be a valuable tool for recognizing LATE, not just in AD but also in LBD patients. This is in line with a recent paper by Wolk *et al*.,^46^ who suggest that including MTA scores could be valuable in defining possible and probable LATE.

In addition to lower right hemisphere amygdalar and hippocampal volumes, we found lower left hemisphere amygdalar volumes in LATE-NC compared with non-LATE-NC donors. However, we found no difference in left hippocampal volumes. These findings can be explained by a possible asymmetry in LATE-NC pathology^47,48^ or the fact that our LATE-NC diagnosis was established on tissue from the right hemisphere. In future endeavours it would be worthwhile to sample the amygdala and hippocampus bilaterally.

Our results show that LATE-NC AD donors had lower amygdalar and hippocampal volumes compared with non-LATE-NC AD donors, but LATE-NC LBD donors only had lower amygdalar volumes compared with non-LATE-NC LBD donors, while we found no difference in hippocampal volume between LATE-NC and non-LATE-NC LBD donors. This could mean within LBD patients, amygdalar volume might be a better indicator of LATE-NC compared with hippocampal volume.

When investigating volumetric differences in amygdalar subnuclei of LATE-NC compared with non-LATE-NC donors, we found that specifically the lateral nucleus was smaller in LATE-NC compared with non-LATE-NC donors. This is partially in line with findings from Makkinejad et al.,^21^ who describe a negative association of TDP-43 and basolateral and superficial nuclei on *ex vivo* MRI volume in LATE-NC donors. The lateral nucleus of the amygdala contains the most connecting fibres to the hippocampus and entorhinal cortex,^49,50^ possibly explaining faster cognitive decline compared with non-LATE-NC donors and explaining the spread of TDP-43 to the hippocampus.

In the hippocampus we found lower dentate gyrus and CA1-4 volumes in LATE-NC donors compared with non-LATE-NC donors. We did not find a significant volumetric difference for the subiculum, even though this region had the highest TDP-43 inclusion load. This is in line with an *ex vivo* MRI study by Wisse *et al*.,^19^ but not with a study by Hanko *et al*.^15^ Furthermore, one study by de Flores *et al*.^18^ reported a selective TDP-43-associated volume loss in the anterior hippocampus. Since we only investigated the middle part of the hippocampus, we are unable to replicate this finding.

Using the available *in vivo* MRI data from our AD cohort, we found that LATE-NC donors showed lower hippocampal volumes compared with non-LATE-NC donors. This is in line with other studies^16,18^ and suggests that lower hippocampal volume can play a role in distinguishing AD from LATE-NC AD in a clinical setting, for example by investigating the rate of hippocampal compared with global atrophy. Unlike the Bejanin *et al*.^16^ study, we did not find a significant difference between LATE-NC AD and non-LATE-NC AD amygdalar volumes. One explanation for this discrepancy could be the low statistical power due to a limited number of available *in vivo* MRIs (*N* = 5) of LATE-NC AD donors. Furthermore, *in vivo* volume loss in the hippocampus might be amplified by co-occuring hippocampal sclerosis.

The distribution of TDP-43 inclusions in the amygdala is relatively uniform across subnuclei, but in the hippocampus we report the highest inclusion density in the subiculum and CA1. Little is known about relative TDP-43 burden in amygdalar subnuclei, but TDP-43 burden in the subiculum, CA1 and dentate gyrus in LATE-NC has been reported in several studies.^12,51,52^ Interestingly, Uemura et al.^12^ report differences in distribution of TDP-43 between LATE-NC AD and LATE-NC LBD in the hippocampus, including a relative increase in TDP-43 burden in the CA2 and CA3 in LATE-NC LBD.^12^ Although we report similar findings (Supplementary Fig. 3), this increase in CA2-3 density is mainly driven by two cases. We report higher amyloid-β and p-tau load in LATE-NC LBD compared with non-LATE-NC LBD donors, which is in line with earlier studies that found higher AD co-pathology in LATE-NC LBD compared with non-LATE-NC LBD individuals.^12,53^ Several studies also found increased Lewy body pathology in LATE-NC AD donors compared with non-LATE-NC AD donors,^2,5^ however, we did not replicate this finding. Nevertheless, in our post-mortem MRI study, our neuropathological findings are based on a single section of the amygdala and hippocampus. Sampling throughout these regions in a larger cohort is recommended to provide a more comprehensive overview of the association between TDP-43 and other pathologies in AD and LBD.

When investigating associations between post-mortem MRI and TDP-43 density in LATE-NC donors, we found that TDP-43 burden predicted amygdalar volume, in line with earlier research.^21^ However, we did not find any significant association between TDP-43 burden and volume in the hippocampus. As previously mentioned, this could be due to TDP-43-associated volume differences seen mostly within the anterior hippocampus,^18^ but also the generally lower hippocampal TDP-43 inclusion density compared with the amygdala. The hippocampus most often is affected at a later LATE-NC stage compared with the amygdala.^29^ Furthermore, measured TDP-43 burden at end stage of the disease might not necessarily reflect LATE-NC severity because severe neuronal loss might lead to reduction of TDP-43 neuronal inclusions.

The main strength of this study is the use of post-mortem *in situ* MRI combined with pathological data of donors with clinically diagnosed and pathological confirmed neurodegenerative disease. However, there are also several limitations to this study. First, due to our relatively small sample size we were unable to investigate differences in association between TDP-43 and volume in AD and LBD separately. The small sample size also affected the power of our *in vivo* MRI analyses. It should also be mentioned that subfield segmentations on 3T T1-weighted MRI have limited accuracy as they are only an approximation of the true subfields.^54^ Lastly, the pathological data was retrospectively obtained from only the right hemisphere. Since LATE-NC can affect both hemispheres at different stages and severity levels,^47,48^ investigating only one hemisphere provides only part of the picture of LATE-NC and its associated volume loss. In conclusion, more research needs to be done on individuals with LATE without neurodegenerative disease and how to distinguish cognitive decline due to LATE from cognitive decline due to AD or types of dementia.

Taken together, our study suggests that TDP-43 pathology in the context of LATE-NC is associated with a 26% lower amygdalar and a 19% lower hippocampal volume in donors with end-stage neurodegenerative disease. TDP-43 burden appears to be an important predictor of amygdalar and hippocampal atrophy, the effects of which can already be observed *in vivo*. Since LATE is associated with a faster disease course and worse cognitive outcomes, these results are relevant towards the development of amygdalar and hippocampal volumes as potential biomarkers for possible LATE in a clinical setting.

## Supplementary Material

awaf201_Supplementary_Data

## Data Availability

Data from the current study are available upon reasonable request.
