# Supplementary material for: Amygdalar and hippocampal volume loss in limbic-predominant age-related TDP-43 encephalopathy
Source: Brain. 2025 Jun 13;148(11):3913–23. doi: 10.1093/brain/awaf201 (PMC12588715; doi:10.1093/brain/awaf201)
Supplement: awaf201_Supplementary_Data [file awaf201_supplementary_data.pdf]

SUPPLEMENTARY FILES

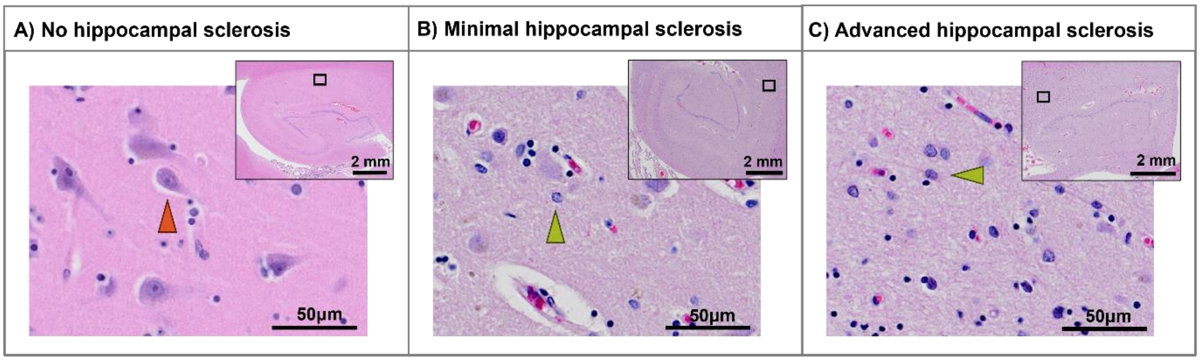

Supplementary figure 1: scoring of hippocampal sclerosis on immunohistochemistry. Hippocampal sclerosis was assessed on haematoxylin and eosin-stained sections of the hippocampus. Hippocampal sclerosis involves gliosis in the CA1/subiculum (green arrows) and the loss of healthy neurons (orange arrow). The donors in our cohort showed either no (n=42, A), minimal (n=6, B) or advanced (n=1, C) hippocampal sclerosis.

Supplementary table 1: specifications of *in-vivo* MRI scans.

ID	Year of death	Year of *in-vivo* scan	Scanner	Dimensions (mm^3)	Resolution (mm^3)	TE (ms)	TR (ms)
**1**	2016	2011	N/a				
**4**	2017	2015	TOSHIBA Titan3T	176x256x256	1	3,4	7,2
**5**	2018	2013	GE3T0 Discovery MR750	176x256x256	1	3,2	8,2
**6**	2018	2016	Philips Achieva	24x288x288	0,76	15	651
**8**	2018	2016	GE3T0 Discovery MR750	176x256x256	1	3,2	8,2
**9**	2019	2014	TOSHIBA Titan3T	176x256x256	1	3,2	9,5
**10**	2019	2013	Philips Ingenuity	192x256x256	1	3	7
**11**	2019	2019	Philips Ingenuity	192x256x256	1	4,5	7,9
**12**	2019	2014	TOSHIBA Titan3T	176x256x256	1	3,2	9,5
**13**	2019	2018	Philips Ingenuity	192x256x256	1	4,5	7,9
**14**	2019	2015	GE3T0 Discovery MR750	176x256x256	1	3,2	8,2
**17**	2022	2021	Siemens MAGNETOM vida	192x256x256	0,9	2,3	2300
**35**	2018	2017	Philips Ingenuity	192x256x256	1	4,5	7,9
**36**	2019	2016	Philips Ingenuity	192x256x256	1	4,5	7,9
**38**	2020	2014	GE3T0 Discovery MR750	176x256x256	1	3,2	8,2
**48**	2022	2019	Philips Ingenuity	192x256x256	1	4,5	7,9
**49**	2022	2017	Philips Ingenuity	192x256x256	1	4,5	7,9

The ID numbers match those in supplementary table 2. Abbreviations: n/a = not available; TE = time to echo, TR = repetition time, ms = milliseconds.

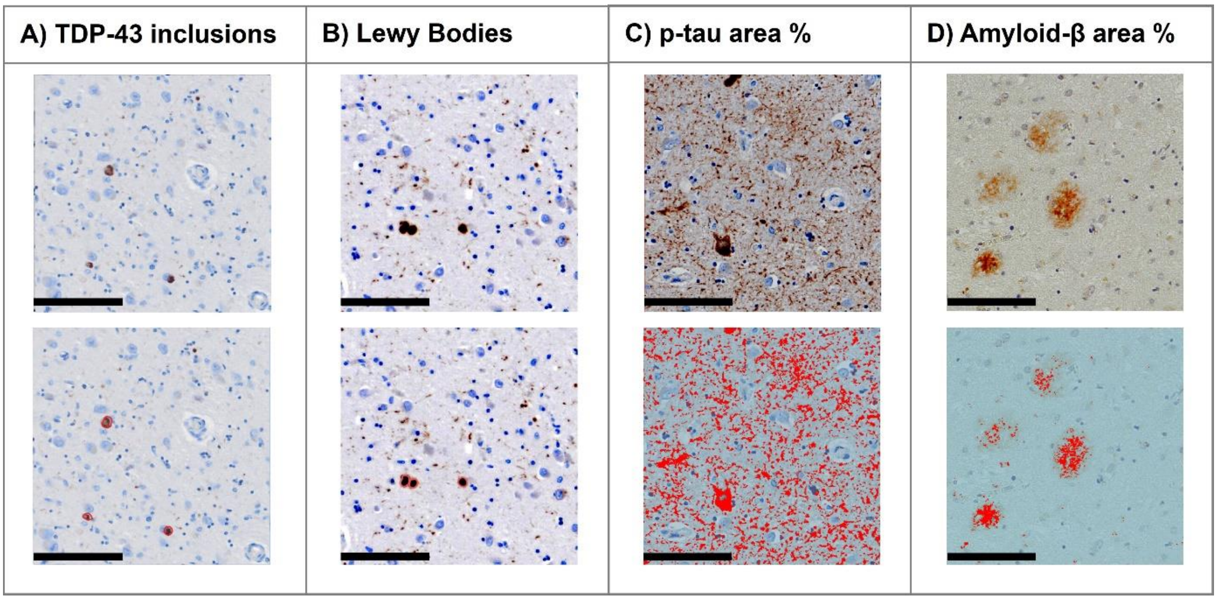

Supplementary figure 2: quantitative measurements of pathological aggregates. The top four images show pathological aggregates of TDP-43 (pTDP-43) (A), a-syn (pSer-129) (B), p-tau (AT8) (C), and amyloid-β (4G8) (D). The bottom four images show detection of positive pixels (p-tau and amyloid-β) or inclusions (TDP-43 and Lewy Bodies) in Qupath[35]. The scale bar represents 100µm for each image.

Supplementary table 2: demographic, radiological and pathological characteristics of donors.

ID	Clinical diagnosis	Sex	Age at death	Disease duration (years)	CDR	MTA	Fazekas	LATE stage	HS	ARTAG	Thal	Braak NFT	Braak LB	APOE
**1**	AD	M	68	6	n/a	1	1	0	0	No	5	5	0	33
**2**	AD	M	69	11	3	3	0	0	0	Yes	5	5	0	34
**3**	AD	F	78	4	3	3	3	0	0	Yes	5	5	3	34
**4**	AD	M	84	13	1	2	1	0	0	No	5	4	0	34
**5**	AD	M	67	9	3	2	0	0	0	No	5	6	0	34
**6**	AD	M	77	4	1	0-1	1	0	0	Yes	5	4	0	34
**7**	AD	M	64	12	3	4	0	0	1	No	5	6	4	34
**8**	AD	F	59	3	3	3	2	0	0	No	5	5	0	34
**9**	AD	M	73	10	3	3	2	0	0	No	5	5	5	34
**10**	AD	M	84	14	n/a	2	2	0	0	Yes	3	4	0	33
**11**	AD	M	65	7	1	1	1	0	0	No	5	5	4	34
**12**	AD	F	72	5	1	2	2	0	0	No	5	6	4	23
**13**	AD	F	60	2	2	1	1	0	0	No	5	6	0	33
**14**	AD	M	68	3	n/a	3-4	2	0	0	No	5	6	0	43
**15**	AD	F	53	7	2	2	3	0	0	No	5	6	0	23
**16**	AD	M	67	10	1	3	0	0	0	No	5	6	4	34
**17**	AD	F	67	1	3	2	1	0	0	No	5	5	0	33
**18**	AD	F	75	n/a	n/a	1	2	0	0	No	5	5	0	43
**19**	PD	F	83	22	n/a	n/a	3	0	0	Yes	0	1	5	n/a
**20**	PD	F	69	15	0,5	2	3	0	0	No	2	2	6	n/a
**21**	PD	F	82	17	n/a	n/a	3	0	0	No	2	2	6	n/a
**22**	PD	M	78	17	0,5	n/a	1	0	0	Yes	1	2	6	n/a
**23**	PD	M	92	16	n/a	2	2	0	0	Yes	3	3	4	n/a
**24**	PD	M	78	20	n/a	1	2	0	0	Yes	1	2	6	n/a
**25**	PD	M	70	8	1	1	2	0	0	No	1	1	6	n/a

**26**	PD	M	93	23	n/a	1	1	0	0	No	2	4	6	n/a
**27**	PD	M	83	n/a	n/a	1	0	0	0	Yes	1	3	6	n/a
**28**	PD	M	85	13	0,5	1	1	0	0	No	1	2	5	n/a
**29**	PD	M	87	19	0,5	1-2	n/a	0	0	No	3	2	6	n/a
**30**	PD	F	93	n/a	n/a	3	3	0	0	No	4	3	0	n/a
**31**	PDD	F	74	12	1	1	3	0	0	Yes	3	2	6	n/a
**32**	PDD	M	79	21	2	1-2	n/a	0	0	Yes	3	2	6	n/a
**33**	PDD	F	81	19	n/a	1	2	0	0	Yes	3	2	6	n/a
**34**	PDD	F	74	n/a	n/a	1	1	0	0	Yes	1	1	6	n/a
**35**	AD	F	80	7	1	2-3	1	2	1	Yes	5	4	0	33
**36**	AD	M	77	10	n/a	1-2	3	2	0	Yes	5	6	0	44
**37**	AD	F	73	1	3	3-4	0	2	0	No	5	6	0	34
**38**	AD	F	61	6	n/a	3	0	2	1	No	5	6	0	44
**39**	AD	M	70	12	n/a	4	3	2	1	No	5	6	4	44
**40**	AD	F	89	7	3	4	3	2	1	No	5	5	5	44
**41**	AD	M	87	n/a	n/a	4	3	2	1	No	5	6	0	43
**42**	DLB	M	91	3	2	1	2	2	0	Yes	3	4	6	n/a
**43**	DLB	F	86	6	3	3-4	3	2	1	No	5	4	6	n/a
**44**	DLB	M	76	n/a	n/a	1	1	2	0	Yes	0	3	6	n/a
**45**	PD	F	86	n/a	n/a	1	2-mrt	2	n/a	No	1	3	6	n/a
**46**	PD	M	84	n/a	n/a	1-2	2	2	0	No	1	3	6	n/a
**47**	PDD	F	83	16	3	3	1	2	0	No	4	4	6	n/a
**48**	AD	M	75	5	3	4	3	3	0	No	5	6	0	44
**49**	AD	M	79	7	n/a	4	3	3	0	No	5	6	2	33
**50**	DLB	M	77	7	3	n/a	n/a	3	0	No	1	3	6	n/a
**51**	PDD	F	94	10	3	2	3	3	n/a	No	4	4	6	n/a

Donors are sorted by LATE stage (0-3) and then by clinical diagnosis. Abbreviations: n/a = not available; AD = Alzheimer's disease; PD = Parkinson's disease; PDD = Parkinson's dementia; DLB = dementia with Lewy Bodies; F = female; M = male; CDR = clinical disease rating; MTA = medial temporal lobe atrophy; HS = hippocampal sclerosis; ARTAG = age-related tau astrogliopathy.

Supplementary table 3: correlations between pathological hallmarks.

			p-tau	Amyloid-β	α -syn
Amygdala	whole amygdala	TDP-43	r=.51 N=11 p=.033	r=.08 N=11 p=.829	r=.42 N = 9 P=.426
		p-tau	x	r=.36 N=29 p=.168	r=.21 N=10 p=.673
		amyloid-β	x	x	r=.15 N=13 p=.625
	Lateral nucleus	TDP-43	r=.30 N=10 p=.452	r=.52 N=10 p=.642	r=.73 N = 8 P=.213 (uncorrected: p=.040)
		p-tau	x	r=.38 N=25 p=.382	r=.67 N=6 p=.673
		amyloid-β	x	x	r=.53 N=9 p=.625
	basal nucleus	TDP-43	r=.30 N=10 p=.486	r=.43 N=10 p=.642	r=.67 N=8 P=.213
		p-tau	x	r=.32 N=25 p=.171	r=.7- N=6 p=.185
		amyloid-β	x	x	r=.51 N=9 p=.315
	accessory basal nucleus	TDP-43	r=.60, N=10 p=.33	r=.08 N=10 p=.829	r=.26 N=8 P=.528
		p-tau	x	**r=.54; p=.030*; N=25**	r=.79 N=6 p=.128
		amyloid-β	x	x	r=.46 N=9 p=.315
	corticomedial nucleus	TDP-43	r=.41 N=10 p=.452	r=.44 N=10 p=.829	r=.20 N=8 P=.426
		p-tau	x	r=.34, N=25 p=.171	r=.79 N=6 p=.128
		amyloid-β	x	x	r=.47 N=9 p=.315
	central nucleus	TDP-43	r=.24 N=10 p=.506	r=-17 N=9 p=.829	r=.47 N=8 P=.528
		p-tau	x	r=.26, N=23 p=.263	r=87, N=6 p=.128 (uncorrected: p=.024)
		amyloid-β	x	x	r=.56 N=7 p=.315
Hippocampus	whole hippocampus	TDP-43	r=.107 N=17 P=.858	r=.43 N=11 p=.375	r=.29 N=8 P=.635
		p-tau	x	**r=.51 p=.003** N=37**	r=.08 N=23 p=.918
		amyloid-β	x	x	r=.1 N=15 p=.779
	dentate gyrus	TDP-43	r=-.05 N=15 P=.858	r=.74 N=11 p=.829	r=-45 N=8 P=.523
		p-tau	x	**r=.71 p=.002** N=35**	r=-.064 N=23 p=.918
		amyloid-β	x	x	r=.124 N=15 p=.779
	CA1	TDP-43	r=.33 N=15 P=.819	r=.55 N=11 p=.280	r=.49 N=8 P=.523
		p-tau	x	**r=.42 p=011* N=35**	r=.29 N=22 p=.686
		amyloid-β	x	x	r=.15 N=15 p=.779
	CA2-3	TDP-43	r=.37 N=15 P=.819	r=.1 N=11 p=.829	r=.25 N=8 P=.636
		p-tau	x	**r=.56 p=.002** N=35**	r=.22 N=22 p=.758
		amyloid-β	x	x	r=-.08 N=15 p=.779
	CA4	TDP-43	r=.52 N=14 P=.858	r=.47 N=11 p=.329	r=-.06 N=8 P=.881
		p-tau	x	**r=.54 p=.002** N=35**	r=-.02 N=22 p=.942
		amyloid-β	x	x	r=.14 N=15 p=.779
	subiculum	TDP-43	r=.084 N=15 P=.858	r=,67 N=11 p=.168 (uncorrected: p=.024)	r=.41 N=8 P=.560
		p-tau	x	**r=.58 p=.002** N=35**	r=,47 N=22 p=.196 (uncorrected: p=.028)
		amyloid-β	x	x	r=.31 N=15 p=.779
	parasubiculum	TDP-43	r=.14 N=14 P=.858	r=.22 N=11 p=.724	r=.63 N=8 P=.523
		p-tau	x	**r=.45 p=.008** N=35**	r=.061 N=22 p=.918
		amyloid-β	x	x	r=.11 N=15 p=.779

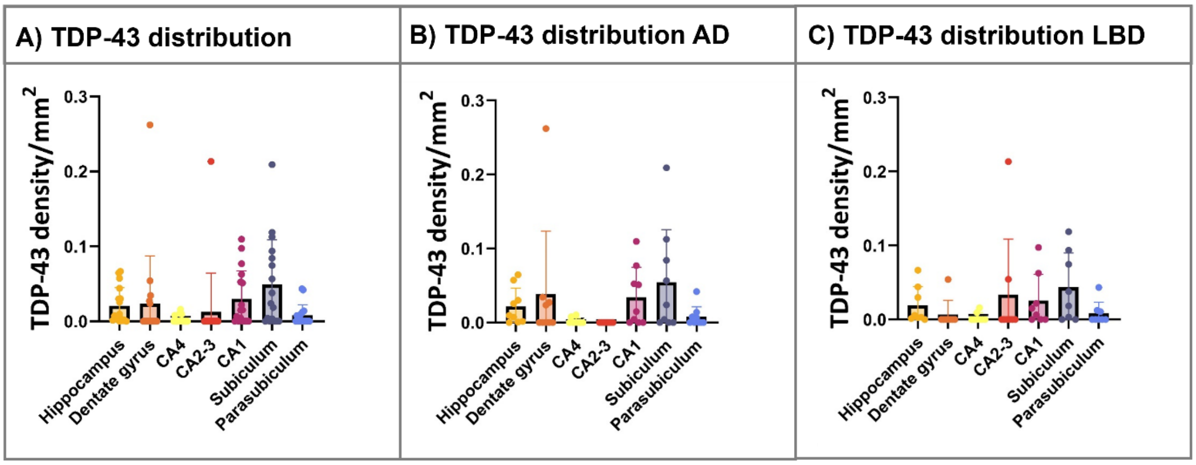

Supplementary figure 3: distribution of TDP-43 inclusions in the hippocampus. The distribution of TDP-43 in the different hippocampal subfields in all (**A**), AD (**B**) and LBD (**C**) donors.

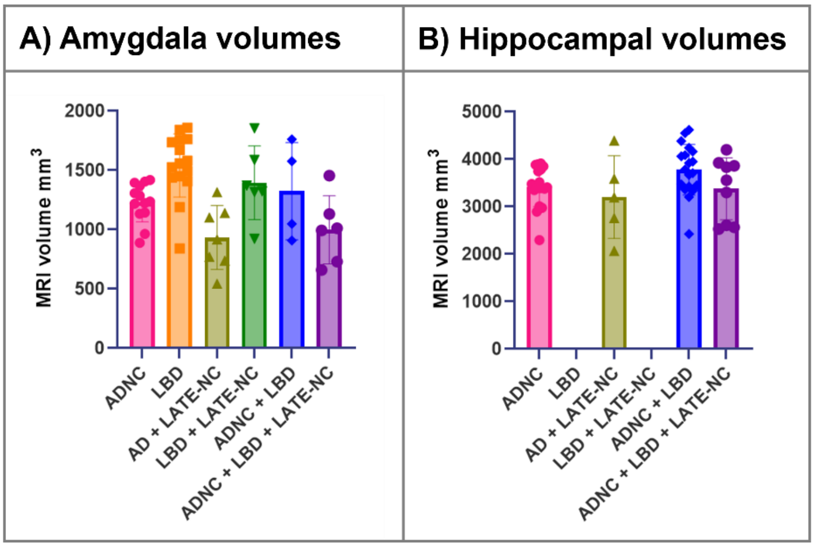

Supplementary figure 4: amygdalar and hippocampal volume of brain donors with different combinations of neuropathologies.
